# A simple technique for quantifying apoptosis in 96-well plates

**DOI:** 10.1186/1472-6750-5-12

**Published:** 2005-05-10

**Authors:** Deborah Ribble, Nathaniel B Goldstein, David A Norris, Yiqun G Shellman

**Affiliations:** 1 UCHSC at Fitzsimons, Dermatology Department, Mail Stop #8127, PO Box 6511, Aurora, CO 80045, USA

## Abstract

**Background:**

Analyzing apoptosis has been an integral component of many biological studies. However, currently available methods for quantifying apoptosis have various limitations including multiple, sometimes cell-damaging steps, the inability to quantify live, necrotic and apoptotic cells at the same time, and non-specific detection (i.e. "false positive"). To overcome the shortcomings of current methods that quantify apoptosis *in vitro *and to take advantage of the 96-well plate format, we present here a modified ethidium bromide and acridine orange (EB/AO) staining assay, which may be performed entirely in a 96-well plate. Our method combines the advantages of the 96-well format and the conventional EB/AO method for apoptotic quantification.

**Results:**

We compared our method and the conventional EB/AO method for quantifying apoptosis of suspension cells (Jurkat) and adherent cells (A375) under normal growth and apoptosis-inducing conditions. We found that our new EB/AO method achieved quantification results comparable to those produced using the conventional EB/AO method for both suspension and adherent cells.

**Conclusion:**

By eliminating the detaching and washing steps, our method drastically reduces the time needed to perform the test, minimizes damage to adherent cells, and decreases the possibility of losing floating cells. Overall, our method is an improvement over the currently available techniques especially for adherent cells.

## Background

Apoptosis, a type of programmed cell death, is an active process. It is a normal component of the development and health of multicellular organisms. The study of apoptosis is an important field of biological inquiry since a deficiency or an excess of apoptosis is one of the causes for cancers, autoimmune disorders, diabetes, Alzheimer's, organ and bone marrow transplant rejection, and many other diseases. Accordingly, a quick and easy assay for quantification of apoptosis would be very useful for many biological researchers.

Currently, methods available to help detect apoptosis *in vitro *include several morphological staining methods (such as ethidium bromide and acridine orange (EB/AO) [1,2], DAPI (4; 6-diamidino-2phenylidole) [2], Hoechst staining [2], and etc), Annexin V staining [3-6], DNA ladder [7,8], TUNEL (Terminal deoxynucleotidyl transferase mediated dUTP Nick End Labeling) [9-11], Caspase-3/7 activity [12-16], and ssDNA staining [17-22]. However, these methods have at least one of the following limitations:

### 1) Involvement of multiple steps

All current staining methods, Annexin V, and DNA laddering assays require detaching, washing and transferring the cells. These procedures might damage the cell membranes and change the cell population distribution of live, apoptotic and/or necrotic cells. Procedures with multiple steps also require more time to perform the assay, and more materials allowing for loss of the cells through the procedures.

### 2) Lack of the ability to quantify live, apoptotic and necrotic cells at the same time

DAPI staining, caspase-3/7 activity, DNA laddering, and ssDNA staining methods only detect increase of apoptotic signals, and can not easily quantify percentage of live, apoptotic, and necrotic cells.

### 3) Non-specific detection

The TUNEL assay is widely used for detecting apoptotic cells. However, it has been shown to provide false positive signals in some necrotic cells [18,21,22].

Despite the many characteristics of apoptotic cells analyzed by current methods, chromatin condensation and nuclear fragmentation remain the hallmarks of apoptotic cells [1,23-25]. It has been suggested that as a rule, classification of cell death in a given model should always include morphological examination coupled with at least one other assay [26]. Fluorescence light microscopy with differential uptake of fluorescent DNA binding dyes (such as EB/AO staining) is a method of choice for its simplicity, rapidity, and accuracy. In such an assay, apoptotic index and cell membrane integrity can be assessed simultaneously and there is no cell fixation step, thus avoiding a number of potential artifacts [26]. Acridine orange (AO) permeates all cells and makes the nuclei appear green. Ethidium bromide (EB) is only taken up by cells when cytoplasmic membrane integrity is lost, and stains the nucleus red. EB also dominates over AO. Thus live cells have a normal green nucleus; early apoptotic cells have bright green nucleus with condensed or fragmented chromatin; late apoptotic cells display condensed and fragmented orange chromatin; cells that have died from direct necrosis have a structurally normal orange nucleus [26].

A 96-well plate format is ideal for examining multiple cell types and performing multiple assays while using very small amounts of materials. It is also a suitable format for high throughput drug screening. To overcome the shortcomings of current methods that quantify apoptosis *in vitro*, and to take advantage of the 96-well plate format, we present here an improved EB/AO staining assay performed entirely in a 96-well plate.

## Results and Discussion

**Figure 1:**
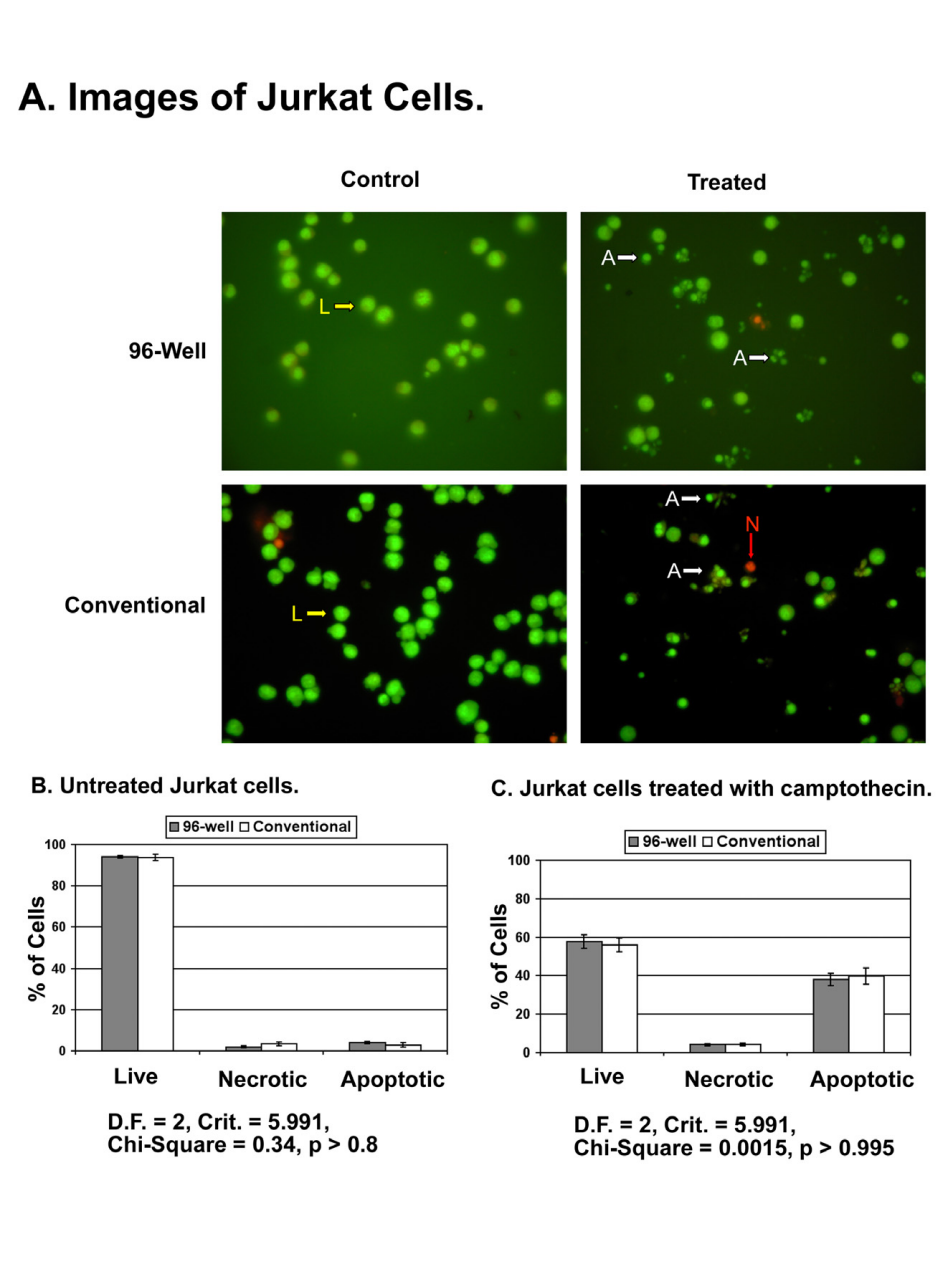
**Comparison of a 96-well-based and the conventional EB/AO method for Jurkat suspension cells. **Jurkat cells were treated with 6 μM camptothecin for induction of apoptosis. (A) -Images of the cells. Yellow arrows next to "L" point to live cells; white arrows next to "A" indicate apoptotic cells; and red arrows next to "N" indicate necrotic cells. (B) Quantification of untreated live, necrotic, and apoptotic cells. (C) Quantification of live, necrotic, and apoptotic cells treated with camptothecin. p-values from chi-square tests were marked on the bottom of each graph.

**Figure 2:**
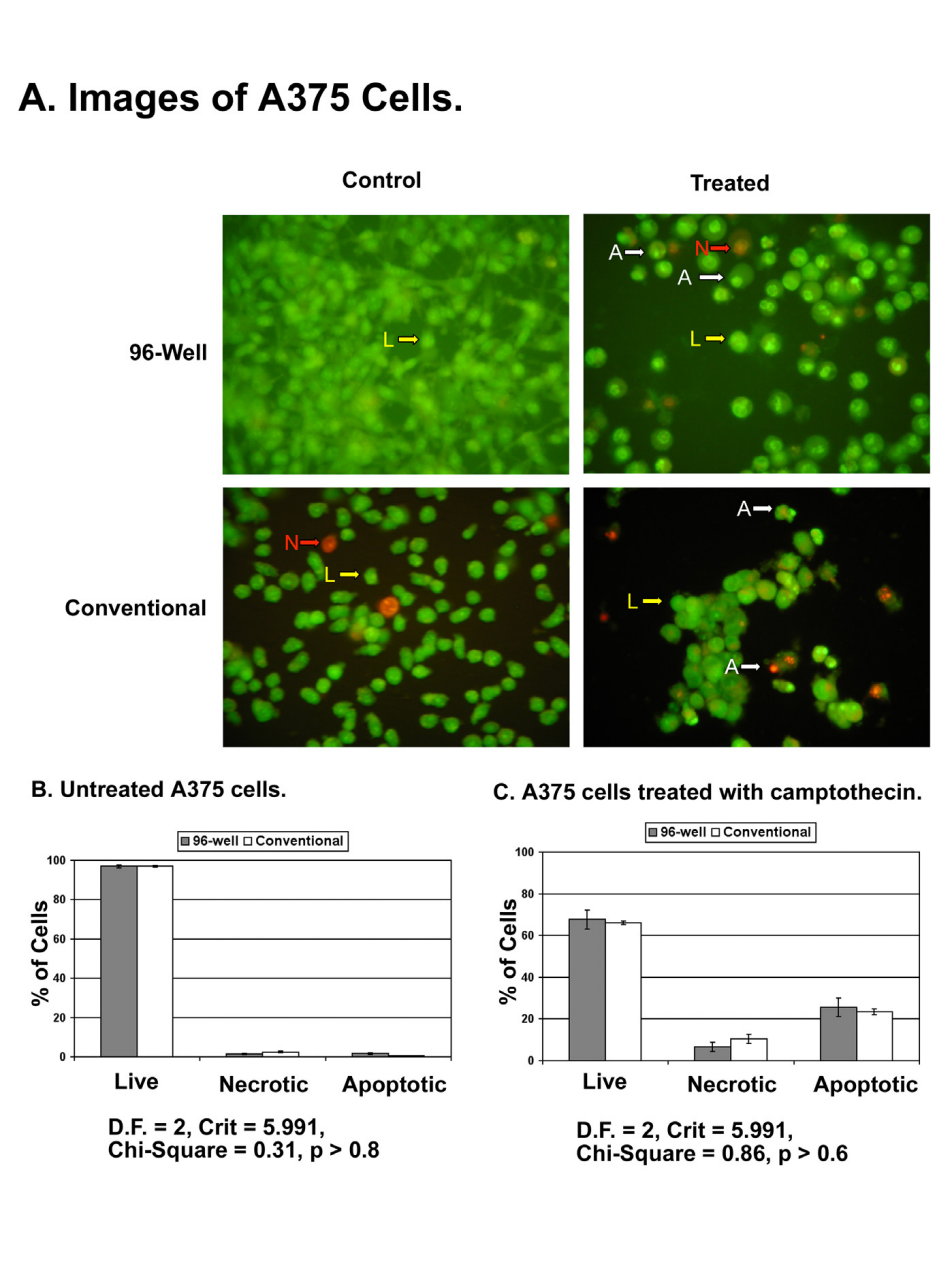
**Comparison of a 96-well-based and the conventional EB/AO method for A375 adherent cells. **A375 cells were treated with 6 μM camptothecin for induction of apoptosis. Symbols and labels are used in the same way as in Figure 1.

We modified the conventional EB/AO method by centrifuging the cells in a 96-well plate to bring down all the cells, including floaters, to the bottom of the plate. Using this method, we obtained quantification of live, necrotic, and apoptotic cells for both suspension cells (Jurkat, Figure 1), and adherent cells (A375, Figure 2), under normal growth and apoptosis-inducing conditions that was comparable to the conventional EB/AO method.

Figure 1 compares the results from our method and the conventional EB/AO methods for both control and treated Jurkat cells. Figure 1A exemplifies cell morphology obtained using both methods. We note that the definition is sharper by eye through the microscope than in the photos. In addition, the pictures obtained using the conventional method, which uses microscope slides, were sharper than those produced using the 96-well based method. This might be due to the relatively poor optical quality of the plastic bottom of 96-well plates. However, this did not encumber the quantification of live, apoptotic, and necrotic cells using the 96-well based method. Further, if high optical clarity is desirable, one may use glass-bottom 96-well plates to eliminate this possible limitation, or use a microscope with Hoffman modulation contrast to increase the optical quality of the plastic [27].

Determining the live, apoptotic and necrotic cells was comparable between these two methods for Jurkat cells: A) live cells have normal nuclei staining which present **green **chromatin with organized structures; B) apoptotic cells contain condensed or fragmented chromatin (green or orange); C) necrotic cells have similar normal nuclei staining as live cells except the chromatin is **orange **instead of green.

Overall, quantification of live, necrotic, and apoptotic control and treated Jurkat cells was quite comparable using both methods (Figure 1B and 1C). The chi-square tests showed that the cell counts were independent of the method used for each treatment condition (For Jurkat Control: Conventional vs. Modified, d.f. = 2, chi-square = 0.34, p > 0.8; For Jurkat Treated: Conventional vs. Modified, d.f. = 2, chi-square = 0.0015, p > 0.995). The largest differences in cell counts between the methods were only a 2% lower live cell count and a 2% higher apoptotic cell count for the treated condition using the conventional method (not statistically significant, Figure 1C). However, these small variations would be within the standard deviations of these assays. Thus, for suspension cells, the modified method was quite similar to the conventional method in terms of quantification and detection of the live, apoptotic, and necrotic cells.

Figure 2 shows the comparison between the modified and conventional methods of staining for A375 adherent cells. Figure 2A presents the morphology of A375 cells from different conditions using the conventional and our 96-well based method. Our method illustrated the morphology of live adherent cells attached to the plate in the control well, and it showed rounded up, detached cells in the treated well. This allowed us to observe more features for distinguishing live cells from apoptotic cells (Figure 2A). In contrast, all the cells stained from the conventional method were rounded up since it requires one to detach the cells. Thus, the modified method allows better morphological assessment of apoptotic condition when compared to the conventional staining technique.

As for Jurkat cells, the quantification of live, necrotic, and apoptotic cell populations in both control and treated A375 cells was quite comparable using both methods (Figure 2B and 2C). The chi-square tests showed that the cell counts were again statistically independent of the staining method used for both treatment conditions (For A375 Control: Conventional vs. Modified, d.f. = 2, chi-square = 0.31, p > 0.8; For A375 Treated: Conventional vs. Modified, d.f. = 2, chi-square = 0.86, p > 0.6). In this case, the largest differences in quantification obtained using these two methods were only 3% necrotic and apoptotic cell counts for treated A375 cells (not statistically significant, Figure 2C). Thus, in accordance with the suspension cell results, the two staining methods were quite comparable for adherent cells. Further, we also found similar results with A375 cells treated with other apoptotic triggers (data not shown).

We note that there was a lot more debris in the adherent cell samples produced using the conventional method. This suggests that the conventional method, with multiple step preparation of adherent cells, could cause some cell damage and break apart some highly fragile cells such as late apoptotic or necrotic cells. This undesired artifact might change the distribution of live, apoptotic, and necrotic cells in the cell population under some conditions.

These results suggest that our method is especially well suited for analyzing adherent cells, since it provides further characteristics to differentiate live cells from apoptotic cells. Additionally, it is much easier to collect all the cells, including floaters, than using the conventional method. Furthermore, by eliminating the detaching and washing steps, this method drastically reduces the time needed to perform the test, decreases the possibility of losing floating cells as in the conventional method, minimizes damage to adherent cells, and may allow more accurate quantification of apoptotic status under certain conditions.

Table 1 summarizes the advantages and disadvantages from various apoptotic detection methods including our 96-well-based EB/AO assay. Our method is the only one with the advantages of having one-step, high specificity, quantification of all three types of cells at the same time, and available in a 96-well format.

## Conclusion

We present here an improved EB/AO staining method by substituting the detaching and washing steps with a simpler centrifugation step with 96-well plates. This modified method combines the advantage of the 96-well format and the conventional EB/AO staining method for apoptotic quantification. Our new technique is easy to perform, time efficient, and especially suitable for adherent cells. In addition, inverted fluorescence microscopes and 96-well plate holders for centrifuges are readily available in most research departments. Our new method only requires small amounts of inexpensive EB and AO for each experiment.

Since this method is straightforward to perform with only one step of gentle centrifugation using 96-well plates, it can also be easily combined with other 96-well-plate-based assays within one experiment, such as cell viability assay (MTS), cell death assay (LDH), or certain caspase activity assays. Therefore, multiple endpoints of cell death and apoptosis can be measured in a single experiment with very small amounts of cells. This could be very valuable for the cells difficult to grow in large amounts (e.g., the short term cultures of patient or animal samples).

In summary, our apoptotic quantification method represents an improvement over the currently available techniques especially for adherent cells. It is highly specific, simple, time efficient, and available in a 96-well format. Further, it can quantify live, apoptotic and necrotic cells at the same time.

## Methods

### Cell lines and normal culture conditions

Both A375 (a human melanoma cell line) and Jurkat (a human leukemia T cell line) were obtained from ATCC (American Type Culture Collection, Manassas, VA, USA). Cells were maintained in RPMI Medium 1640 (Invitrogen, Grand Island, New York, USA) with 10% fetal bovine serum (Gemini Bio-Products, Inc. CA, USA) and grown in an incubator at 37°C with 5% CO_2_. All tissue culture plates and other plastic wares were from Costar (Corning, New York, USA).

### Reagents

Camptothecin was purchased from Biovision (Mountain View, CA, USA). Acridine orange (AO), ethidium bromide (EB), and ethylenediamine-tetraacetic acid (EDTA) were purchased from Sigma (St. Louis, MO, USA).

The dye mix for the EB/AO staining was 100 μg/ml acridine orange and 100 μg/ml ethidium bromide in PBS [2].

### Induction of apoptosis in Jurkat cells with camptothecin treatment

Four ml of 5 × 10^5 ^cells/well were seeded in a 6-well plate the night before the treatment. Cells were treated with camptothecin at a final concentration of 6 μM for 4 hr in the 37°C incubator before the cells were subjected to EB/AO staining. Then, 1 ml of cell suspension was used for conventional EB/AO staining, and 100 μl of cell suspension was transferred to a 96-well plate for modified EB/AO staining.

### Induction of apoptosis in A375 cells with camptothecin treatment

For the conventional EB/AO method, 2.5 × 10^5 ^cells/well were seeded in a 6-well plate the night before the treatment. For our modified EB/AO method, 1.6 × 10^4 ^cells/well were seeded in a 96-well plate. Cells were treated with camptothecin at a final concentration of 6 μM for 48 hr in the incubator before the cells were subjected to EB/AO staining procedure.

### Conventional EB/AO staining

Procedures were followed as described previously in Current Protocols of Immunology [1]. Briefly, cells were harvested as the following: 1) For suspension cells, 1 ml of Jurkat cell suspensions was transferred to a 15 ml tube. 2) For adherent cells, supernatant (medium and floating A375 cells) were transferred to 15 ml tubes. The rest of the adherent cells were detached with PBS-EDTA, Dulbecco's phosphate buffered saline (Invitrogen, Grand Island, New York, USA) containing 1 mM EDTA. The supernatant and the detached cells from the same sample were pooled together in the 15 ml tubes.

Both Jurkat and A375 cells were pelleted by centrifuged at 1,000 RPM (129 g) for 5 minutes using a Beckman Model TJ-6 centrifuge, and washed with 1 ml of cold PBS once. Cell pellets were then re-suspended in 25 μl cold PBS and 2 μl EB/AO dye mix was added. Stained cell suspension (10 μl) were placed on a clean microscope slide and covered with a coverslip. Cells were viewed and counted using a Nikon eclipse TS100 inverted microscope at 400× magnification with excitation filter 480/30 nm; dichromatic mirror cut-on 505 nm LP; and barrier filter 535/40 nm (Melville, NY, USA). Pictures were taken with a Nikon COLPIX digital camera. Tests were done in triplicate, counting a minimum of 100 total cells each.

### 96-well-based EB/AO staining

For both suspension and adherent cells, 96-well plates were centrifuged at 1,000 RPM (129 g) for 5 minutes using a Beckman Model TJ-6 centrifuge with inserts for 96-well plates. EB/AO dye mix (8 μl) was added to each well, and cells were viewed under the same microscope as above. Tests were done in triplicate, counting a minimum of 100 total cells each.

### Statistical analysis

To test the hypothesis that frequencies of observed live, necrotic, and apoptotic cells were independent of the staining method used, we employed chi-square tests for independence in contingency tables using Microsoft Excel. For each contingency table, triplicate counts of live, necrotic, and apoptotic cells for both the traditional and modified staining methods were averaged.

## Authors' contributions

YGS conceived the study, participated in its design and coordination, and drafted the manuscript. DR participated in experiment design, carried out the experiments, and helped with the first draft of the manuscript. NBG performed the statistical analysis and final editing of the manuscript. DAN provided the general guidance and research funding for the study. All authors read and approved the final manuscript.

## Figures and Tables

**Table 1:** Comparison of the advantages of various apoptotic detection methods.

**ASSAY**	**ONE-STEP**	**QUANTIFICATION OF LIVE, NECROTIC AND APOPTOTIC CELLS**	**SPECIFICITY**	**96-WELL FORMAT**
Our 96-well-based EB/AO	+	+	+	+
Caspase 3/7 Activity	+	-	+	+
Conventional EB/AO	-	+	+	-
Annexin V/PI	-	+	+	-
ssDNA	-	-	+	+
DNA Laddering	-	-	+	-
TUNEL	-	-	-	+
DAPI stain	-	-	+	-
